# Preliminary testing of eye gaze interfaces for controlling a haptic system intended to support play in children with physical impairments: Attentive versus explicit interfaces

**DOI:** 10.1177/20556683221079694

**Published:** 2022-02-28

**Authors:** Javier L. Castellanos-Cruz, María F. Gómez-Medina, Mahdi Tavakoli, Patrick Pilarski, Kim D Adams

**Affiliations:** 1 Faculty of Rehabilitation Medicine, University of Alberta, Edmonton, Canada; 2 Electrical & Computer Engineering Department, University of Alberta, Edmonton, Canada; 3 Department of Medicine, University of Alberta, Edmonton, Canada

**Keywords:** Attentive user interface, children, eye gaze interface, explicit eye input interface, haptic guidance, play, prediction

## Abstract

**Introduction:**

Children with physical impairments may face challenges to play because of their motor impairments, which could lead to negative impacts in their development. The objective of this article was to compare two eye gaze interfaces that identified the desired toy a user wanted to reach with a haptic-enabled telerobotic system in a play activity.

**Methods:**

One of the interfaces was an attentive user interface predicted the toy that children wanted to reach by observing where they incidentally focused their gaze. The other was an explicit eye input interface determined the toy after the child dwelled for 500 ms on a selection point. Five typically developing children, an adult with cerebral palsy (CP) and a child with CP participated in this study. They controlled the robotic system to play a whack-a-mole game.

**Results:**

The prediction accuracy of the attentive interface was higher than 89% in average, for all participants. All participants did the activity faster with the attentive interface than with the explicit interface.

**Conclusions:**

Overall, the attentive interface was faster and easier to use, especially for children. Children needed constant prompting and were not 100% successful at using the explicit interface.

## Introduction

Play is critical for children to develop the skills needed to assume student, family, and social roles throughout their lives.^1^ Play is a way for children to expand their knowledge about self and the world and allows them to discover and enhance their capabilities by trying out objects, making decisions, understanding cause-and-effect relationships, and seeing the consequences of their actions. However, children with physical impairments may face challenges to play and explore their environment and this may negatively affect their social, emotional, and/or psychological development.^2^

Robots can allow children with physical impairments to explore and interact with their environment, which can contribute to their learning and social development.^3^ Robots can be teleoperated, allowing children to be able to use an interface mounted on their wheelchair to control an environment-side robot to manipulate toys.

Haptics-enabled robots can provide several benefits for robot control. Haptic interfaces can provide the sense of touch to the children, so that they can learn about the properties (e.g. hardness or softness) of their toys.^4^ Haptics-enabled robots can also implement kinesthetic haptic guidance, or haptic guidance for short, for helping individuals with physical impairments to more accurately control the robots. Haptic interfaces can apply haptic guidance in the form of artificial potential fields, that is, applying forces in the direction of a location or along a trajectory. Artificial potential fields have been used to improve handwriting in children with cerebral palsy (CP) using a pen-like haptic robot to follow the templates given as guidelines.^5^ If the child’s handwriting was off the template, force feedback was provided to pull the child’s hand toward and along the trajectory of the character. Artificial potential fields have also been used to help control the speed and direction of a powered wheelchair, applying forces to the joystick when the child went off the path,^6^ and to help children avoid obstacles while they try to reach a location.^7^ Attracting forces were applied on the joystick to follow the path, while repelling forces were applied when the wheelchair got close to an obstacle.

Another form of haptic guidance is known as forbidden region virtual fixtures (FRVF), which is accomplished by the generation of forces to keep the robot end-effector inside or outside a pre-defined space. For instance, FRVF were created in the form of figure shapes such as circles or squares to support coloring.^8^ Forbidden region virtual fixtures served as guides for the users to reduce the amount of area colored outside the given templates. FRVF can also be helpful in tasks such as sorting, by guiding users from one location to another, for example, along a cylindrical space.^9^ Forbidden region virtual fixtures are intended to reduce the time and area covered by individual’s movements, and reduce unintended movements produced by spasticity in people with physical impairments.

If haptic guidance is to be used to help children move towards a target, the system needs to know what the desired target is. Eye gaze is a logical method to indicate that desire. A common eye gaze approach is to use an explicit eye input interface, which requires the users to control their eye movements, or gaze direction, voluntarily and consciously, for example, using the eyes as a pointer to choose targets or movement commands.^10^ However, explicit interfaces can be difficult for children with disabilities to use.

The literature is scarce at describing and measuring the performance of children when they use eye gaze interfaces, making it difficult to determine at what age children should be able to successfully use explicit eye gaze interfaces.^11^ Children with physical impairments were capable of gazing or dwelling at an object on a computer screen for more than one second.^12^ Children as young as 9 months of age were capable of dwelling their gaze on a target to select it with about 80% success rate, and the rate improve to 100% at 11 months.^13^ However, the performance on that task cannot generalize to more complex tasks that include multiple objects.

Children with physical impairments may have less success using explicit eye gaze systems than children without physical impairments. Children with CP between 7 and 11 years old took longer to maintain their gaze at a target image for a dwell time than typically developing children between 4 and 13 years old.^14^ Children with CP took about four times longer than typically developing children to maintain their gaze on the target image, on average. Children with CP had difficulties maintaining their gaze on the target due to their body movements. In another study, children with Rett syndrome (a developmental disorder involving cognitive and neuromotor impairments) between 4 and 9 years of age were capable of selecting the correct picture with their gaze only 62.4% of the time, on average.^15^

Children may find it difficult to use explicit interfaces while completing a task that requires multiple steps. Encarnação et al.^16^ tested an explicit interface to control a Lego robot with three children with CP of 3 and 6 years of age. Children controlled the movement of the robot by looking at a computer screen that displayed symbols to move the robot forward or backward and turn left or right. Children had to fixate on the screen to select and then look at the robot to observe its action. The 3-year-olds were not able to complete the activities, and the authors suggested it was due to the complexity of changing the focus of their attention from the screen to the robot. In another study, a 7-year-old child without impairments was not able to use an explicit eye input interface for drawing on a computer screen.^17^ The interface required users to fixate at a location on the canvas or buttons on the screen for at least 500 ms to set the starting point for drawing, selecting a shape, or setting the end point of the selected figure. Other participants, who were between 10 and 36 years of age, were able to use the interface successfully.

Most of the literature of eye gaze interfaces is about explicit interfaces (pointing or fixating at targets on a screen) rather than attentive user interfaces, which could be easier to use for children since they do not require explicit actions. Attentive interfaces can be a form of intelligent interfaces that track and process the user’s point of gaze (POG) to provide information about the user’s attentional behavior while performing a task.^10^ Li et al.^18^ implemented a visual attention recognition method to control a laparoscope during minimally invasive surgery. The system had an attentive user interface that autonomously controlled the motorized laparoscope to the site where the surgeon’s visual attention was directed. Barbuceanu et al.^19^ designed an attentive interface to identify the user’s intentions for object selection in a virtual environment. The user’s eye gaze was first analyzed during the selection of objects in a virtual kitchen. The interface incorporated a probability model of the gaze transitions between the objects, representing the possible operations to perform with them, for example, pour water from a bottle into a glass. Establishing such connections between the objects allowed the system to anticipate the user’s selection of the objects. The literature around attentive user interfaces is limited, and the use of this technology has not been explored with children. The present study had the purpose of comparing an attentive user interface with an explicit eye input interface when these activated the haptic guidance of a telerobotic haptic system towards the desired toy. The difficulties children and individuals with physical impairments faced using these interfaces were also examined.

## Methods

The technology developed in this study is intended to be used by children with physical impairments, however, the technology is at a testing stage. In this preliminary study we included one adult and child with cerebral palsy (CP) to gain insight about user experience and ensuring the safety of our technology before the target population use it. Explicit and attentive interfaces were designed in order to explore and gain insight of performance and demands of eye gaze interfaces.

### Participants

For this preliminary stage, a convenience sample of participants was recruited through announcements to parents on listservs in the city of Edmonton, Canada. Five typically developing children participated in this study. Their ages ranged from 3 years and 11 months to 4 years and 10 months (52.8 ± 3.9 months). There were three females and two males. None of the children had any known physical or visual impairments. Also, a 52-year-old female adult who has quadriplegic cerebral palsy participated in this study. She has difficulties handling objects due to poor motor control and spastic movements. Additionally, a child with hemiplegic cerebral palsy participated in this study. He was 7 years and 4 months old. His right limbs are affected, and he has difficulties grasping and reaching objects with his right hand. More details about the child and the adult with CP can be found in Castellanos-Cruz JL et al.^20^

Consent was obtained from the children’s parents and verbal assent was obtained from the children prior to starting the trials. The adult provided consent for her participation. Ethical approval was obtained from the University of Alberta.

### Materials

**Figure 1.**
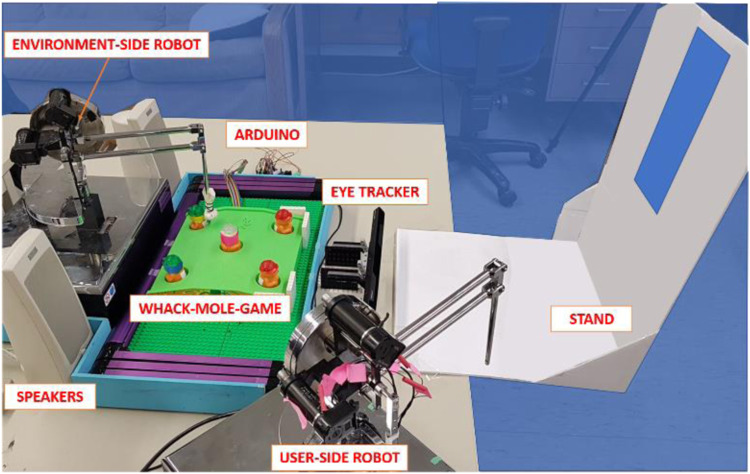
Experimental setup of the robotic system and the game. Children sat behind the stand and looked through the hole. The stand was not in place for the adult with CP. Figure adapted from Castellanos-Cruz JL et al. .^21^

Figure 1 illustrates the setup of the robotic system and the activity. The robotic system had two PHANToM Premium 1.5 A haptic robots (3D Systems, Inc., Rock Hill, SC, USA), one of them was placed as the environment-side robot and followed the movements performed by the user on the other robot, the user-side robot. The system also included a Tobii EyeX eye tracking system (Tobii Technology, Stockholm, Sweden) to measure the x and y coordinates of the point of gaze POG. More details about the system can be found in Castellanos-Cruz JL et al.^21^

The activity chosen for this study was a whack-a-mole game. Children looked through the hole of a stand, to prevent the eye tracker from losing the view of the eyes due to head movements. The stand was not in place for the adult with CP because it interfered with her wheelchair. The distance between the participants’ eyes and the eye tracker was approximately 65 cm. For more details, see Castellanos-Cruz JL et al.^20^

### Haptic guidance

**Figure 2.**
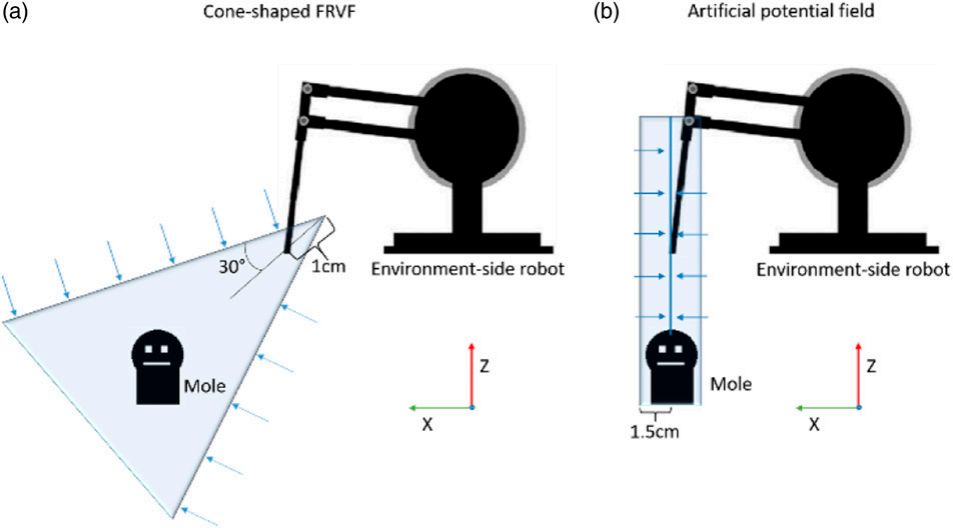
2D projection of the haptic guidance: (a) 3D cone-shaped FRVF for guiding the user towards moles. (b) 3D artificial potential field for helping the users to whack the moles. Figure adapted from Castellanos-Cruz JL et al.^21^

The haptic guidance was developed because of observations in Castellanos-Cruz JL et al.^20^ There were two types of haptic guidance: a cone-shaped FRVF and an artificial potential field, as illustrated in Figure 2. The cone-shaped guidance (Figure 2(a)) was designed to help the user reach the moles. It allowed the user to move the robot end-effector closer to the target mole and prevented the user from moving further away from it. The cone had a 30-degree angle and there was 1 cm of space between the robot’s end-effector and the cone’s origin. The potential field guidance (Figure 2(b)) was implemented to help the user whack the moles once the environment-side robot’s end-effector was close to them. When the robot’s end-effector was 1.5 cm away in the XY plane from the target mole the potential field guidance attracted the robot end-effector toward a virtual vertical line that passed through the mole. This way it helped the user to push straight down on the mole. Forces were applied toward the x and y coordinates of the target mole, to help them center over top of it, but forces were not applied in the z-axis so that the user could move the end-effector up and down on their own.

### Eye gaze interfaces

#### Attentive user interface

The attentive interface was implemented as determined in Castellanos-Cruz JL et al.^20^ First, the distances between the user’s POG and each mole were measured, then the mole with the least distance was assigned as the predicted mole, and the guidance was directed toward it.

#### Explicit eye input interface

**Figure 3.**
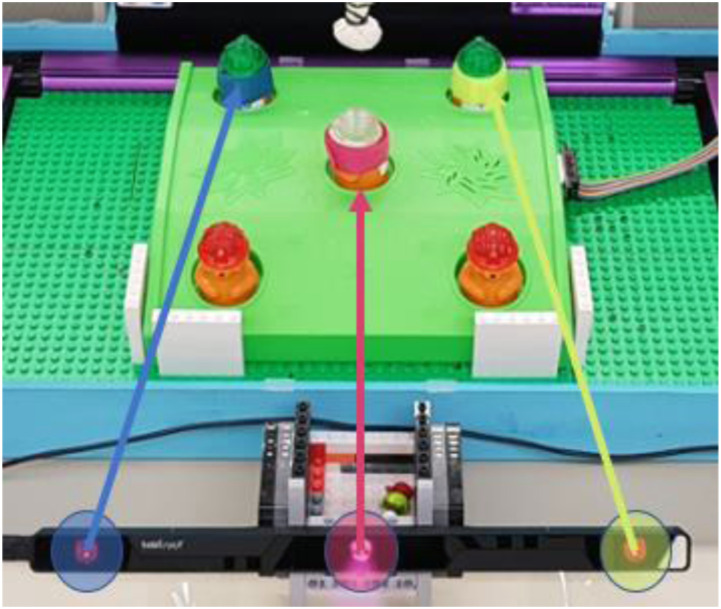
Illustration of the dwell-spot of the explicit eye input interface, which corresponded to each mole. The blue mole is on the left, the pink mole is in the middle, and the yellow mole is on the right.

An explicit interface was designed with dwell locations corresponding to the moles in the game. Each of the three lights in the eye tracker corresponded to each of the three target moles in the game. Fixating at a light for a minimum of 500 ms activated the guidance towards the respective mole. Once a mole was selected the computer spoke out loud the mole ID (i.e. blue, pink, or yellow) to let the participants know what mole he/she had selected. Figure 3 illustrates how each dwell-spot on the eye tracker corresponded to each mole. The design of this interface was based on the literature of explicit eye gaze interfaces, where all studies with children^11–17^ included a computer screen. In this study, the eye tracker itself played the role of the screen with dwell-spots. The time of 500 ms was chosen based on the study by Hornof A, Cavender A, and Hoselton R^17^ and previous studies^10^ where the dwell time ranged from 300 ms for experienced users to 1000 ms for novices. The dwell time plays a key role to overcome the Midas Touch problem, which refers to the problem of unintentional eye gaze selections.^10^

### Procedure

There was one session which consisted of two parts with a five-minute break in-between. The session took between 20 and 40 min. The typically developing children controlled the robot with their dominant hand, the adult with CP used her left hand (her choice), and the child with CP controlled the robot with his affected hand, the right hand.

#### Eye tracker calibration

To calibrate the eye tracker, all participants were asked to fixate at each dwell-spots (the lights in the eye tracker), or each mole in the game, for one second, this was repeated five times. In the case of children, the stand played a role to avoid large head movements that would cause the calibration to fail. In the case of the adult, she held a constant pose throughout the sessions, and the stand was not necessary.

#### Part 1 – Testing the attentive user interface

This part was focused on testing the attentive user interface while it activated the haptic guidance. It was carried out using an experimental crossover design, comparing two conditions: when the guidance was activated by the interface (“with guidance”) and not having the guidance (“without guidance”). The attentive interface activated only the artificial potential field guidance for the children (since they only required haptic guidance to whack the moles, not to go toward them). In the case of the adult participant, the attentive interface also activated the cone-shaped guidance (the guidance depicted in Figure 2(a)) to help her reach the moles. Before starting the activity, the eye tracker was calibrated with respect to the three moles.

All participants whacked 54 moles in total during the game. The 54 moles were divided into six sets of nine moles, in which the conditions “with guidance” or “without guidance” were alternated, with the first condition randomly assigned for each participant. Participants did three sets in the “with guidance” condition and three in the “without guidance” condition. For the adult with CP, a short break was given between sets to ask her whether the most recent set was easier than the previous set, but she was not told if guidance was on or off. At the end of the trial, she was asked if her eyes felt tired. Her responses were recorded by the researcher into the research notes.

#### Part 2 – Testing the explicit eye input interface

This part had the purpose of testing the explicit eye input interface while it activated the haptic guidance. It only had a “with guidance” condition in order to compare it to the “with guidance” condition of the attentive interface. The explicit interface activated only the artificial potential field guidance for the children, and both the cone-shaped guidance and artificial potential field for the adult, as in part 1.

Before starting the activity, an explanation of how to control the robot and the explicit interface was given to the all participants. To get familiar with how the explicit interface worked, all participants whacked 10 moles that were lit up randomly. In the case of the children, researchers pointed out the spots where they had to fixate (eye tracker’s lights), and then showed them that the system said the color of the mole they had selected. The children were asked to whack the mole and to feel the haptic guidance. After whacking the mole, they were shown that the haptic guidance would not let them move towards other moles unless they fixated at the dwell-spots that corresponded to the other moles, and this was because the potential field guidance above the chosen mole was activated. After familiarization, the children’s understanding of the interface was tested by asking them where they had to look if they wanted to whack each mole, for example, “if you want to whack the blue mole, where do you look?”

For the experiment, participants whacked 45 moles using the explicit interface. Prompting to look at the dwell-spots was given to the children if they tried to go to the lit-up mole by overcoming the potential field haptic guidance without first fixating at a dwell-spot. At the end of this part, the adult with CP was asked if her eyes felt tired and which interface she preferred and the reasons why. Her responses were recorded into the research notes.

### Data collection and analysis

The participants’ POG and the environment-side robot’s position were recorded, and both parts of the session were video recorded. After the session, the POG and robot’s position data were synchronized and divided into episodes. Episodes were excluded when the eye gaze was lost due to head movements or the robot’s force limits were exceeded.

In part 1 with the attentive interface 124 episodes were included for the typically developing children, 23 for the adult with CP, and 22 for the child with CP in the “without guidance” condition. In the “with guidance condition,” 124 episodes were included for the typically developing children, 22 for the adult with CP, and 21 for the child with CP.

The prediction accuracy was measured as the percentage of time in which the output of the attentive interface corresponded to the target mole. The prediction accuracy was measured after the participants’ POG was closer to the target mole than the other two moles.

The episodes of each condition were processed after the session to obtain the time the participants took to whack each mole and the distance traveled by the end-effector of the robot. For the adult with CP, the jerkiness of the movements was measured using the Log Dimensionless Jerk (LDLJ) measure.^22^ This jerkiness measure was used to examine how the adult’s movements were affected by the haptic guidance: the lower the value of LDLJ the jerkier the movements.

The results of time, distance, and jerkiness were compared between the “without guidance” and the “with guidance” conditions. Linear mixed-effects models were used for statically comparing the results obtained from the typically developing children. Independent t-test was applied for the statistical comparisons of the results obtained from the child and the adult with CP, separately. Both statistical tests were performed using a 95% confidence level.

In part 2 with the explicit interface, episodes where the participants were prompted were excluded. The remaining episodes were 200 for the typically developing children, 19 for the child with CP, and 35 for the adult with CP. From these episodes the success rate of using the explicit interface for selecting the correct moles was calculated. Success rate was measured as the percentage of episodes in which the participants fixated at the dwell-spot that corresponded to the lit-up target mole.

The time the participants took to whack each mole and the distance traveled by the robot’s end-effector was calculated in each episode. For the adult with CP, the jerkiness of her movements was measured using LDLJ.

Results of time, distance, and jerkiness were compared between the attentive and explicit interfaces. Linear mixed-effects models were used for statically comparing the results obtained with the typically developing children. Independent t-test was applied for the statistical comparisons of the results obtained with the child and the adult with CP, separately. Finally, the body and head movements that the participants did when using both eye gaze interfaces were compared by observing the videos from part 1 and part 2 of the study.

## Results

In part 1 with the attentive interface, all the participants were able to control the robots to whack all moles without help. During the “with guidance” condition, the accuracy of the predictions of the attentive interface was 96.06% (SD = 8.48) for the typically developing children, 89.03% (SD = 15.51) for the adult with CP, and 94.65% (SD = 9.8) for the child with CP. The adult with CP commented that the “without guidance” and “with guidance” conditions had the same difficulty. In the “with guidance” condition, she felt that the cone-shaped haptic guidance was sometimes against her movements when she was trying to reach the target mole, but that the guidance of the artificial potential field was helpful to whack the moles.

Table 1 lists the average times and distances traveled to whack each mole, during the “without guidance” and “with guidance” conditions for the attentive interface. The statistical differences between with and without guidance that were significant are marked with an asterisk. Jerkiness for the adult with CP was −21.48 ± 2.5 without and −20.56 ± 2.05 with guidance.

**Table 1.** Statistical comparisons of the “without guidance” and “with guidance” conditions when participants used the attentive user interface.

	Guidance	TDC	Child CP	Adult CP
Time (s)	Without	1.35 ± 0.40*	2.04 ± 1.03	2.64 ± 1.47
	With	1.53 ± 0.33*	1.80 ± 0.77	2.40 ± 1.22
Distance (m)	Without	0.23 ± 0.04	0.65 ± 0.25	0.62 ± 0.33
	With	0.23 ± 0.03	0.63 ± 0.27	0.52 ± 0.25

TDC – Typically developing children.

*Statistical difference between without and with guidance (*p*-value < 0.05).

Table 2 lists the percentage of episodes where the participants required prompting and the success rate of using the explicit interface to select the correct moles in Part 2. None of the children changed their selection when it was wrong. They moved towards the mole as soon as the computer spoke out the color of a mole, even if they selected the wrong mole. In addition, children tried to move toward the other moles before gazing at the dwell-spots, however, the haptic guidance did not allow them.

**Table 2.** Percentage of episodes that participants required prompting and success rate of using the explicit eye input interface for selecting the correct target moles.

Participant	Age (months)	Percentage of episodes that required prompting (%)	Success rate (%)
TDC1	47	18.37	85
TDC2	52	5.13	89.19
TDC3	53	0	90
TDC4	54	0	80
TDC5	58	43.9	65.22
Child with CP	88	52.50	63.16

TDC: Typically developing children.

Table 3 lists the average times and distances traveled of the movements when the participants used the attentive and explicit interfaces in the “with guidance” condition (note that the results of the attentive interface listed in Table 3 are the same as those listed in Table 1 for the “with guidance” condition). Jerkiness for the adult with CP was −20.56 ± 2.05 with the attentive interface and −20.28 ± 1.97 with the explicit interface. The adult with CP commented that the guidance activated by the explicit interface was helpful to reach and whack the moles when using the explicit interface, and she did not feel the forces were against her movements, as with the attentive interface. She felt that her eyes were tired after using the explicit interface, unlike when using the attentive interface. Overall, she said she preferred using the attentive interface because it was not tiring, and it was easier and faster than using the explicit interface.

**Table 3.** Statistical comparisons of the attentive user interface and the explicit eye input interface, both with guidance.

	Interface	TDC	Child CP	Adult CP
Time (s)	Attentive	1.53 ± 0.33*	1.80 ± 0.77*	2.40 ± 1.22*
	Explicit	3.79 ± 1.89*	3.70 ± 1.50*	5.61 ± 2.08*
Distance (m)	Attentive	0.23 ± 0.03*	0.63 ± 0.27	0.52 ± 0.25
	Explicit	0.38 ± 0.19*	0.81 ± 0.34	0.61 ± 0.32

TDC: Typically developing children.

*Statistical difference between without and with guidance (*p*-value < 0.05).

From the videos, it was observed that all children moved their trunk and heads more when using the explicit interface than when they used the attentive interface. All children turned and inclined their heads in the direction of the dwell-spots to select the respective moles, which was unnecessary given the distance they were from the eye tracker. In the case of the adult with CP, she also inclined her head instead of just moving the eyes and sometimes this caused the eye tracker to lose the view of her eye, therefore, she had to lift her head up for the system to be able to record her POG.

## Discussion

According to Table 1, when using the attentive user interface typically developing children spent significantly more time to whack the moles when the guidance was activated (“with guidance” condition) than when it was not (“without guidance” condition). This was unexpected, as we thought the guidance should improve their performance, but examination of the children’s eye gaze and the environment-side robot’s position during both conditions revealed that children moved the robot towards the target mole while they were still looking at the previous mole. Thus, in the “with guidance” condition the guidance was still activated towards the previous mole, preventing them from moving towards the target mole, causing the increase of time to whack the moles.

In the case of the participants with CP, they spent less time to whack the moles when the guidance was activated, although, it was not statistically significant, which may be due to the low sample size. The child with CP experienced difficulties grasping the user-side robot’s end-effector tightly and he could not whack the moles as hard as the typically developing children. The artificial potential field guidance in the “with guidance” condition made it easier for him to whack the moles because the potential field guidance helped him to keep the robot over top of the mole so that he just had to push downwards. The potential field guidance also helped the adult with CP to whack the moles when she experienced spastic movements while trying to hit them. However, she did not think that the cone-shaped FVRF was helpful to reach the moles because the guidance opposed her movements sometimes when it predicted the wrong mole.

There was no significant difference between the distance traveled by the robot’s end-effector when the participants played the game in the “without guidance” and the “with guidance” conditions. However, the distance traveled by the adult with CP was about 10 cm less with the guidance than without the guidance. One possible reason is that the haptic guidance helped to reduce the range of her spastic movements, and her movements were less jerky with the guidance than without it. However, it was not statistically significant, possibly due to the low sample size.

The attentive user interface achieved high accuracies of 96.06% and 94.65% when the children with and without physical impairments used it. The interface did not reach 100% because the children’s visual attention was possibly sometimes on the environment-side robot’s end-effector, rather than always on the target mole. The accuracy of the interface was lower for the adult with CP, 89.03%, and a possible reason is that she experienced involuntary movements that might have driven her visual attention away from the target mole to the environment-side robot’s end-effector, and in those instances the attentive interface would have made the guidance go towards the closest mole to the end-effector. This likely contributed to her comment that the guidance was sometimes against her movements to reach the moles.

Children had some difficulties using the explicit eye input interface despite the interface only requiring one step to operate it. Three out of five typically developing children required promoting to look at the dwell-spots in 5.13–43.90% of the episodes, and the child with CP needed prompting in 52.5% of the episodes. When prompting was not required, typically developing children used the explicit interface to select the correct target moles with a success rate between 65.22% and 90%, and the child with CP’s success rate was 63.16%. Children were still able to whack the correct moles despite selecting the wrong moles with eye gaze. The reason that they could whack the moles was because the potential field guidance was only activated when the robot’s end-effector was less than 1.5 cm away from the selected mole (correct or wrong). For example, if they had selected the pink mole in the middle of Figure 3 but the target mole was the blue mole (left), they could whack the blue target mole as long as they did not get close to the pink mole (1.5 cm). This was why none of the children corrected their eye gaze selections when they were wrong. If the cone-shaped guidance had been activated for the children, then they would possibly have corrected their selections, because the cone-shaped guidance would have guided them to the selected (non-desired) mole. In the case of the adult with CP, she did not have trouble understanding how to use the explicit interface, thus she did not need prompting and her success rate was 100%.

With the explicit interface, children had to transition their gaze between the play area and the dwell-spots, and they also had to think about which dwell-spot corresponded to the mole that was lit up and think about moving the robot. With the attentive interface, children only had to think about controlling the robot to complete the activity. Considering that the literature on eye tracking technology with children usually includes a computer screen, it is necessary for future work to test explicit interfaces with dwell-spots within the environment and on desired toys. This way the complexity of the explicit interface can be reduced.

The explicit interface not only required the users to change their visual behavior (i.e. maintaining focus on the dwell-spots) but it also changed the way they moved their head and trunk. All participants moved their heads in the direction of the dwell-spots, and sometimes this caused the eye tracker to fail at measuring their POG. Movement of their head and trunk can affect the calibration of the eye tracker or can cause the eyes to be outside the eye tracker’s workspace.

Participants spent more time when using the explicit interface than the attentive interface. Of course, the length of the dwell took time, but children also took additional time to remember that they had to fixate at the dwell-spots, and the adult with CP took additional time because she had to adjust the position of her head for the system to be able to record her POG.

On average, all participants traveled longer distances to whack the moles using the explicit interface than when using the attentive interface. However, statistical significance was only achieved for the results of the typically developing children. The longer distance with the explicit interface was due to the movements the participants made with the robot while fixating at the dwell-spots. The children were constantly moving while they were trying to overcome the haptic guidance to go to the target mole. The adult with CP moved the robot while fixating at the dwell-spots because she could not keep the robot’s end-effector completely steady due to involuntary movements.

In terms of jerkiness, there was no statistical difference between the jerkiness of the movements of the adult with CP while using the explicit and attentive interfaces. However, she mentioned that the guidance was more helpful while using the explicit interface than the attentive interface. The reason was likely because once she selected a mole, the haptic guidance applied by the explicit interface was directed to towards the selected mole the entire time and did not oppose her movements as it sometimes did with the attentive interface.

The accurate guidance with the explicit interface was at the cost of spending more time and traveling longer distances. Additionally, the adult experienced tiredness in her eyes when using the explicit interface, whereas she did not feel tiredness in her eyes when using the attentive interface. Overall, the adult with CP said she preferred the attentive interface because it was easier and faster to use.

The usability of the eye gaze interfaces may have been affected because we used a stationary eye tracker. With this eye tracking system, the user had to be near the eye tracker, and when the user moved or inclined her/his head, re-calibration was required, or the eye tracker lost the view of the eyes. However, these limitations could be addressed by using a wearable eye tracker, if children would tolerate it.

The attentive and explicit interfaces may not work in all activities. The attentive interface may not work in other activities where there are more toys or where the 3D POG (i.e. x, y coordinates and depth of the POG) is required to discriminate between objects that are at different distances but in the same line of vision. Also, the attentive interface may not work as well as it did in this study if the objects or toys are closer to each other. The complexity of the explicit eye input interface may increase and lead to different results if there are more objects in the environment because more dwell-spots would need to be added.

Limitations of this study include the sample size which does not allow to generalize these results to all children, nor to different types of cerebral palsy or physical impairments. Comparisons should not be made between participants in this study, since results may be highly individualized. The comparisons between the two eye gaze interfaces may be biased because all participants tested the attentive interface first and then the explicit interface. In the case of the child with CP, his performance at using both interfaces may have been affected by his attention deficit disorder. Also, this study had only a single session, and results could improve as the participants gain more experience with the robotic system and the interfaces.

For future work, it will be necessary to recruit more children, and test the interfaces in other activities and with a wearable eye tracker. Additionally, it will be necessary to consider the prevalence of visual difficulties in children with physical impairments, and to investigate the conditions for which eye gaze based robotic systems could be useful and effective.

## Conclusions

This study showed preliminary testing results with typically developing children, and one child and one adult with CP, allowing us to gain insight about technology performance and user experience controlling a haptic system with eye gaze. It showed that the eyes can be used to predict with high accuracy what object individuals want to reach with a telerobotic haptic system. An attentive user interface can be implemented to apply haptic guidance towards the predicted object without requiring the user to focus for a period of time at the target object as required with explicit eye input interfaces. Though the explicit interface tested in this study could be made more efficient (e.g. dwell-spots in the environment, lower dwell time), the attentive interface implemented provided advantages over the explicit interface, such as lower times to complete the activity and less distances traveled by the robot. Typically developing children and the child with CP were not 100% successful at using the explicit eye input interface and required prompting to use it. The adult participant with spastic CP perceived that the attentive interface was easier and faster to control the robotic system than with the explicit interface. The attentive interface activated the guidance according to the user’s eye-robot coordination for the robotic system of this study, and this likely contributes to a more natural and intuitive interaction between the user, the robot, and the environment than the explicit interface, which required the participants to gaze off the environment to select a toy. However, the guidance was not always what the user intended, so further research is needed to improve the predictions before testing with more children who have CP.
